# Tenofovir-Associated Kidney Dysfunction and Bone Fracture: A Case Report and Literature Review

**DOI:** 10.7759/cureus.61562

**Published:** 2024-06-03

**Authors:** KK Athish, Shobhana Nayak Rao, Venkat H Marimuthu, Vamsi Krishna, Anwadevi Arun

**Affiliations:** 1 Department of Internal Medicine, Sri Devaraj Urs Academy of Higher Education and Research, Kolar, IND; 2 Department of Nephrology, Sri Devaraj Urs Academy of Higher Education and Research, Kolar, IND; 3 Department of Emergency Medicine, Sri Devaraj Urs Academy of Higher Education and Research, Kolar, IND

**Keywords:** human immunodeficiency virus (hiv), highly active antiretroviral therapy (haart), avascular necrosis of femur, avascular necrosis of the hip, femur intertrochanteric fracture, tenofovir alafenamide (taf), drug-induced acute renal failure, kidney failure, acquired immune deficiency syndrome (aids), tenofovir disoproxil fumarate (tdf)

## Abstract

Tenofovir is an integral part of antiretroviral therapy used to treat HIV. Long-term use of tenofovir has been associated with decreased glomerular filtration rate, leading to chronic kidney disease, as well as acidosis, electrolyte imbalances, and tubular dysfunction. Tenofovir can also disrupt bone health by decreasing renal phosphate absorption, contributing to osteomalacia. This leads to disruption in mineral metabolism, elevated parathyroid hormone levels, and ultimately, low bone mineral density. Replacing tenofovir with alternative antiretroviral therapy can improve kidney function if done early in the course of the disease. Here, we discuss a case of a 65-year-old woman with HIV who presented with advanced renal failure and hypophosphatemia-induced bone fracture attributed to long-term use of tenofovir. We conclude monitoring kidney function and considering alternative antiretroviral therapy is important to prevent and manage these side effects in patients on long-term tenofovir therapy.

## Introduction

Despite global initiatives to contain human immunodeficiency virus (HIV) spread and progression, the disease remains a significant public health challenge in resource-limited and developing countries. Highly active antiretroviral therapy (HAART) has considerably decreased morbidity and mortality among HIV-positive patients [1]. Tenofovir disoproxil fumarate (TDF) and tenofovir alafenamide fumarate (TAF) are among the first-line drugs in the antiretroviral regimen for HIV infection and are integral components of HAART [2,3]. While TDF offers a convenient dosing schedule, good efficacy, and a minimal initial side-effect profile, it has been linked to over half of renal tubulopathies in HIV-infected patients [2,3]. Furthermore, TDF can cause bone toxicity, characterized by osteopenia, osteomalacia, and fractures [2-4]. A case series by Fioroti et al. [2] has reported a combination of renal and bone toxicity, similar to our study. Here, we present a case of a patient with renal failure and osteomalacia-induced bone fracture associated with TDF use. The case is presented due to the paucity of these two complications of long-term tenofovir in the published literature.

## Case presentation

A 65-year-old woman living with HIV presented to the emergency room with progressive breathlessness at rest for two weeks duration. She had been on HAART for seven years and was taking a TLD (tenofovir, lamotrigine, and dolutegravir) regimen. In the past two days, she also developed bilateral lower limb pain. She reported a history of diffuse bone pain, particularly intense in her right hip for the past six months. This pain had worsened significantly in the last week. There was no history of falls, trauma, or other significant medical conditions. She had attained menopause 15 years back. Her vital signs on initial examination were as follows: pulse rate of 96 beats/minute, blood pressure of 130/90 mmHg, room air oxygen saturation of 92%, and respiratory rate of 24 breaths per minute. On auscultation, bilateral basal crackles were heard.

**Figure 1:**
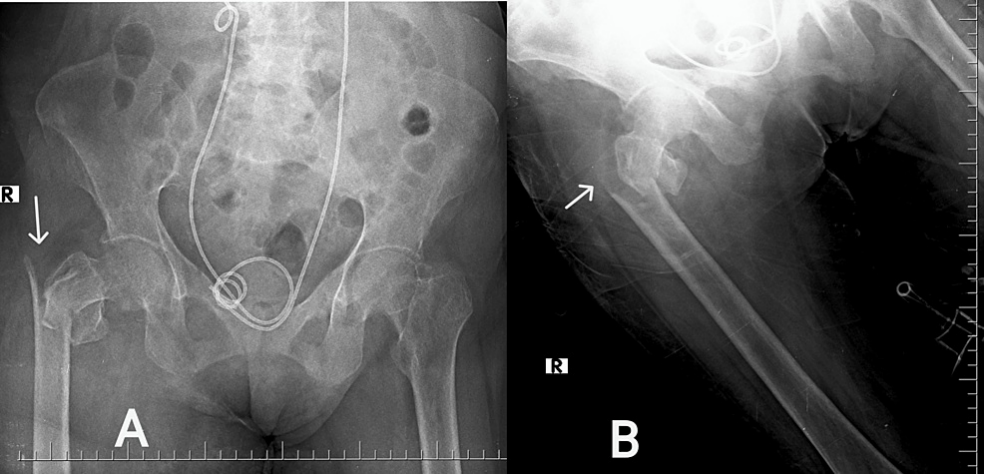
Plain X-ray of the matured skeleton. (A) Anterior-posterior (AP) view of the pelvis with bilateral hip and (B) AP view of the right femur revealing displaced comminuted intertrochanteric fracture (white arrow) of the right femur (Boyd and Griffin’s type 2).

Examinations of the cardiovascular, central nervous system, and abdomen were normal. Examination of the lower limbs revealed external rotation and mild flexion of the right leg, with tenderness and restricted movement in the right hip joint. Arterial blood gas analysis revealed high anion gap metabolic acidosis with a 2.8 mmol/L lactate. Chest X-ray was normal, while a radiograph of the bilateral hip revealed a partially displaced fracture of the right intertrochanteric region (Figure 1). Investigations are mentioned in Table 1.

**Table 1:** Laboratory parameters.

Serum parameters	Reference range	At admission	After treatment
Hemoglobin (g/dl)	11.5-15	6.3	8.9
White blood count (/µL)	5000-11000	16800	8739
Platelet (/µL)	150-300	284	265
Blood urea (mg/dl)	15-40	125	78
Creatinine (mg/dl)	0.6-1.1	8.9	5.6
Sodium (mEq/L)	136-145	128	139
Potassium (mEq/L)	3.5-5	4.4	4.7
Erythrocyte sedimentation rate (mm/hr)	Less than 20	130	83
CD4 count (cells/mm^3^)	500-1500	371	-
D-dimer (ng/mL)	Less than 500	452	-
Procalcitonin (ng/ml)	Less than 0.1	2.7	-
Parathyroid hormone (pg/ml)	10-60	1315	503
Inorganic phosphorus (mg/dl)	2.5-4.5	4.6	4
Calcium (mg/dl)	8.5-10.5	6	8.9
Magnesium (mg/dl)	1.7-2.2	1.9	1.7

She tested negative for hepatitis B, hepatitis C, and rheumatoid factor. Urinalysis revealed 3+ red blood cells, 2+ protein, and 10-16 white blood cells per high-power field. Liver function tests were within normal limits, but her estimated glomerular filtration rate (eGFR) was less than 10 ml/min. After stabilizing the patient with intravenous antibiotics, nebulization, and hemodialysis (HD), open reduction and internal fixation with a dynamic hip screw of the fracture were performed under spinal anesthesia following adequate HD and blood transfusion. Her antiretroviral therapy (ART) regimen was switched to a triple-drug combination of abacavir, lamivudine, and ritonavir. She also received adequate nutritional supplementation and regular maintenance HD. On a follow-up visit after three months, she was found to be stable, continuing HD along with high-dose calcium, vitamin D (cholecalciferol), and bisphosphonate (35 mg/week of alendronate sodium) to treat osteoporosis. Secondary hyperparathyroidism with parathyroid hormone (PTH) levels reduced to 500 pg/ml, with complete remission of acidosis, hypophosphatemia, and hypouricemia throughout her outpatient follow-up. However, her renal functions did not improve significantly for her to discontinue hemodialysis and she remains on thrice weekly dialysis.

## Discussion

Effect on kidney

The kidney exhibits an age-related decline in some structure and function. In older adults, the reduction in glomerular filtration rate (GFR) or creatinine clearance (CrCl) is roughly 1.0 mL/min per 1.73 m^2^ each year, while a yearly decline in GFR is about 1% after the third decade [5]. A study by Ryom et al. [6], involving over 10,000 HIV-positive individuals, found that with yearly TDF exposure, the risk of having chronic kidney disease rose by 34%. According to the European guidelines, patients on TDF with eGFR less than 60 mL/minute should be offered an alternate regimen if their eGFR falls by 5 mL/year for three consecutive years or more than 25% from the baseline. National Institutes of Health (NIH) guidelines recommend cautious use or avoidance of TDF in patients with existing kidney impairment [7].

Tenofovir, a nucleotide analog reverse transcriptase inhibitor, inhibits retrovirus replication. It is excreted through glomerular filtration and proximal tubular secretion of the kidneys. Tenofovir can suppress DNA polymerase activity at high intracellular concentrations, leading to mitochondrial dysfunction. Active uptake causes chronic renal tubular damage and a decrease in eGFR [8,9]. TDF shows no interaction with cytochrome P-450 [9]. However, TDF toxicity has been linked to polymorphism in the multidrug-resistant protein (MRP) coding gene, which may affect drug excretion [10]. Proximal convoluted tubule (PCT) damage from nucleoside analogs can lead to persistent hypophosphatemia, which can cause osteomalacia and inadequate mineralization of the bone matrix. Furthermore, proximal tubule dysfunction might decrease vitamin D hydroxylation in the proximal tubules [8]. In this context, moderate to severe proximal tubular dysfunction may precede bone toxicity.

Over the past two decades, TDF has emerged as a crucial element of HIV therapy. TDF was initially considered safe for kidney function in the short to medium term; however, it can cause a small, consistent decrease in eGFR [9]. Manifestations like metabolic acidosis, varying degrees of bicarbonate, and phosphaturia indicate mild to major tubular injury [9]. PCT is responsible for secreting hydrogen ions, synthesizing calcitriol, and reabsorbing glucose, small protein molecules, including β2-microglobulin and vitamin D-binding protein (VDBP), uric acid, phosphate, and amino acids [9,11]. Long-term TDF use causing serious tubular damage has been linked to Fanconi syndrome, a dysfunction of the PCT that impairs the reabsorption of these essential molecules from the urine back into the blood, leading to aminoaciduria, glucosuria, and severe osteomalacia. Consequently, these molecules undergo renal excretion, resulting in severe hypophosphatemia and osteomalacia due to insufficient bone mineralization [9,11,12].

A multivariate analysis of post-marketing safety data for TDF identified several risk factors for increased creatinine levels during TDF therapy: low body weight, advanced age, elevated pre-treatment creatinine, longer treatment duration, and lower CD4 count [3,13]. Concomitant use of high-dose nonsteroidal anti-inflammatory drugs and other nephrotoxic agents with TDF should be avoided due to the potential for additive renal dysfunction [3].

Mechanisms of bone abnormalities

TDF treatment can worsen the effects of proximal tubulopathy on bone metabolism by causing hyperparathyroidism and accelerated bone turnover. It increases PTH levels and VDBP, which decreases free 1, 25-OH(2)D (calcitriol). This leads to an excess of PTH and reduced calcium absorption. Increased plasma TDF levels result in lower free 1, 25-hydroxy-vitamin D levels [14-16]. Furthermore, vitamin D3 supplementation may have a therapeutic impact when TDF-containing ART reduces bone mineral density (BMD) and attenuates elevation of PTH levels [15,17]. In a randomized controlled trial conducted among 441 patients by Martin et al. [18], comparing tenofovir-emtricitabine (TDF-FTC) and abacavir-lamivudine (ABC-3TC) at weeks 48 and 96, the former group demonstrated markedly decreased hip and spine t scores and had developed skeletal lesions such as osteopenia or osteoporosis or received antiresorptive therapy, while among the latter group, a rise in both t scores was noted. A higher incidence rate of osteonecrosis of the femur has been reported in retropositive patients, which is around 100 times higher than the anticipated incidence in the general population [7].

Replacing tenofovir with other suitable ART medications is the most effective way to manage tenofovir-induced nephrotoxicity. Withdrawal of TDF can lead to an improvement in kidney function. However, this recovery may be slow and not complete, especially in patients whose eGFR is declining gradually or those not taking a protease inhibitor [19]. Studies have shown that after stopping tenofovir due to acute kidney injury, around half of patients regained normal kidney function within a follow-up period of approximately two years. However, one case required hemodialysis for four months [20]. Our patient too did not recover kidney function enough and is dialysis dependent. These findings highlight the debate surrounding the severity of tenofovir's side effects and its long-term safety.

## Conclusions

Tenofovir can cause various kidney problems, including acute kidney injury, chronic kidney disease, proximal tubular cell damage, and Fanconi syndrome. Therefore, in patients on long-term tenofovir, close monitoring of renal parameters and eGFR is crucial to prevent nephrotoxicity. Withdrawing the drug may be the treatment approach for long-term irreversible renal damage. While kidney function typically improves after stopping tenofovir, this improvement may be incomplete. Early intervention, including potentially replacing tenofovir with an alternate regimen, may lead to better kidney function in the long term.
